# Mental Health Status Among the Staff of Harm Reduction Centers

**DOI:** 10.5812/ijhrba.12244

**Published:** 2014-03-05

**Authors:** Majid Rezazade, Zeynab Lashani, Khodabakhsh Ahmadi

**Affiliations:** 1 AIDS Prevention and Control Committee, Welfare Organization State, Tehran, IR Iran; 2 Department of Human Sciences, Shahed University, Tehran, IR Iran; 3 Behavioral Sciences Research Center, Baqiyatallah University of Medical Sciences, Tehran, IR Iran

**Keywords:** Mental Health, Harm Reduction, Anxiety, Depression, Substance-Related Disorders

## Abstract

**Background:**

Creating a supportive environment encourages charity services to help risk groups and individuals which has magnificent impacts on reducing their harm.

**Objectives:**

According to this plan, the purpose of this study was to investigate the mental health status in the staff of harm reduction centers.

**Materials and Methods:**

The clustered sample of this comparative study consisted of 49 staff of harm reduction centers. The study was supported by the United Nations Development Program in Tehran, Iran. The participants completed GHQ-28 and DASS-21 questionnaires along with sociologic forms and the results were evaluated by descriptive statistics indexes and independent sample t-test.

**Results:**

One-hundred percent of the participants in this study showed the symptoms of psychological disorders, and approximately 16 percent suffered from moderate to high degree of anxiety, depression and stress. The level of anxiety (P ˂ 0.04) and stress (P ˂ 0.01) in the younger staff (less than 40 years) was significantly higher than older staff (more than 40 years old). In addition, somatic symptoms (P ˂ 0.05) and social withdrawal (P ˂ 0.01) were significantly higher in women than men.

**Conclusions:**

Accordingly, major mental disorders in the staff of harm reduction centers, especially women and younger people need to be considered more than before.

## 1. Background

In 2004, the World Health Organization (WHO) suggested to perform developed interventions for harm reduction to prevent HIV outbreak in all countries. One of the harm reduction programs in Iran is run jointly by AIDS Prevention and Control Committee, Welfare Organization State, Behavioral Sciences Research Center, Baqiyatallah University of Medical Sciences and backed by the financial support of global fund to fight aids, tuberculosis and malaria (GFATM) and United Nations Development Programme (UNDP). In these centers HIV/AIDS prevention education is provided for people with high risk behaviors such as intravenous drug use or unprotected sex. Harm reduction among high risk behavior people with HIV/AIDS is one of the preventive strategies to reduce unhealthy impacts on the society and economy. Harm reduction interventions include educating the people with high risk behavior and providing them with syringes, condoms, and alternative treatments for opioids and so on (1, 2). Regarding disorders and problems of high risk behavior people (3-5) these interventions are of great importance and are applicable in many situations (6).

Effective approaches to reduce harms are as follows:

- Promoting health actions through applying the general policies of health promotion and AIDS prevention.
- Creating a supportive environment for people at risk and/or with high risk behaviors.
- Familiarizing health centers with AIDS prevention services and principles.
- Contributing actively in the developing and strengthening personal skills in decision making.
- Communicating with others, adopting assertive decision and changing high risk behaviors (7).

Therefore, creating a supportive environment ( for the people at risk, and/or with the high risk behavior) to build trust and provide services have a significant effect on HIV prevalence reduction (7); a significant part of this important task is the responsibility of the staff of harm reduction centers.

But, one of the main problems of addicts is communicating with non-addicts who live with them (8), so dealing with high risk people may cause problems such as unsustainability in the staff of harm reduction centers. Since mental health is one of the variables which improves jobs sustainability (9) and associates with internal power resources, it can increase the capability of the staff, despite unfavorable conditions and negative events (10). Stresses, unsuitable living conditions, social exclusions and employment disparities are associated with mental health disorders. Besides, stresses and coping skills of individuals are among important factors associated with mental health problems (11).

Since mental health is a significant factor in preventing social problems such as addiction to alcohol, drugs, and gambling (12), the current study has investigated the mental health of staff of Tehran harm reduction centers as one of the effective variables of harm reduction. Thereby, the question is: “How well is the mental health status of staff of the harm reduction center?”

## 2. Objectives

According to this plan, the purpose of this study was to investigate the mental health status in the staff of harm reduction centers.

## 3. Materials and Methods

In the current comparative study, the subjects were the staff of harm reduction centers of UNDP Global Fund project in Iran selected by cluster sampling method: 49 subjects including 34 (69.4%) men and 15 (30.6%) women. The centers were as follows: Shosh, Fallah, Rey-Nafarabad, Rey-Sarcheshme, Khaniabad, Valiasr Town, Jannatabad, Ostad Moein and Sadeghieh. 

Data collection tools were general health questionnaire (GHQ-28), and depression, anxiety and stress scale (DASS-21). This questionnaire was designed by Goldberg and Hillier in 1979 and is the most widely used tool for general screening in mental health which included 28 questions with Likert scoring as A = 0, B = 1, C = 2, and D = 3 (13). The validity of this questionnaire is reported 95% by Goldberg and Williams (14) and has been widely used as a major screening method in more than 30 countries such as Brazil, Chili, Germany, France, Greece, India, Italy and Japan (14).

Gibbons et al. identified four factors including physical symptoms, anxiety, insomnia, social functioning disorder, and depression among a student population sample (15). The highest correlation was observed among physical symptoms, anxiety and insomnia. Reliability of the questionnaire was measured by test-retest with 0.74 reliability coefficient, 6.7 cut-off point, 0.88 test accuracy, and 84.2 specificity (15).

In the current study, according to the Fathiashtiani and Dastani (16) and Yaghoubi (17), the cut-off point was considered as 7 points for subscales, and 23 points for total scale which indicates the level of disorder. The scale of depression, anxiety and stress (DASS-21) was designed by Lovibond and Lovibond and is a combination of three self-assessment subscales to measure the negative emotional state of depression, anxiety and stress (18). Each subscale has 7 items.

Validity and reliability of this scale have been evaluated in Iranian population by Asghari Moghaddam et al. (19). They confirmed the exploratory factor analysis of three-factor structure of DASS, and also the reliability of its scales through evaluating internal consistency and retest indexes. Generally, the validity and reliability of the tests were satisfactory.

The correlation between DASS depression subscale and Beck’s depression scale was 0.69, and between DASS depression subscale and anxiety subscale, four-system anxiety, was 0.62. In factor analysis results, the three-factor model (depression, anxiety and stress) was able to explain the data better than other models. Generally, three-factor model explained 54.04% of variance.

 In the current study, according to Fathiashtiani and Dastani DASS-21 cut-off points are higher than 10.5 for depression subscale, higher than 7.5 for anxiety subscale, and higher than 13 for stress subscale which showed the higher level of the disorder (16).

The results of statistical descriptive tests and t test in independent groups were evaluated by statistical package for the social science version 18.

## 4. Results 

Descriptive results from demographic features indicated that 49% of subjects were single, 46.9% married, and 2% divorced. Also, 12.5% had a bachelor or higher degrees, 8.2% associate degree, 26.5% high school diploma and 38.13% lower degrees (Table 1). 

**Table 1.** Demographic Features of the Staff of Harm Reduction Centers

Variable	No. (%)
**Gender**	
Men	34 (69.4)
Women	15 (30.6)
**Marital status**	
Single	24 (49)
Married	23 (46.9)
Divorced	1 (2)
**Education**	
Illiterate	2 (1.4)
Elementary	3 (6.1)
Guidance school	8 (16.3)
High school	7 (14.3)
High school Diploma	13 (26.5)
Associate degree	4 (8.2)
Bachelor and higher	12 (24.5)

Analyzing descriptive indexes of depression, anxiety and stress (DASS-21) scores of staff of harm reduction centers showed that 16% suffered from severe and higher level of anxiety, 16.3% had severe and higher level of depression, and 16.3% with severe and higher level of stress. Also, according to the GHQ questionnaire, 100% of the subjects suffered from some symptoms of psychological disorders, such as physical disorders, anxiety, socio-occupational disorders and depression (Table 2). 

**Table 2.** Descriptive Indexes of Anxiety, Depression and Stress in the Staff of Harm Reduction Centers

	Average	Standard Deviation	Cut-off Point	People With Disorders, No. (%)
**Anxiety (DASS)**	3.20	3.84	7.5	8 (16)
**Depression (DASS)**	6.33	4.27	10.5	8 (16.3)
**Stress (DASS)**	5.34	5.33	13	8 (16.3)
**Physical disorder and insomnia (GHQ)**	11.03	3.63	7	49 (100)
**Anxiety disorder (GHQ)**	11.87	4.25	7	49 (100)
**Socio-occupational Disorder (GHQ)**	12.75	3.13	7	49 (100)
**Severe depression (GHQ)**	9.11	3.71	7	49 (100)
**General health disorder (GHQ)**	45.18	12.35	23	49 (100)

Comparing the level of anxiety, depression, stress and general health among the staff of harm reduction centers, according to the demographic features showed that the level of anxiety (P ˂ 0.04) and stress (P ˂ 0.01) among the staff younger than 40 were significantly higher than those of over 40. Level of physical disorders and insomnia (P ˂ 0.05), and socio-occupational disorders (P ˂ 0.01) in women staff of harm reduction centers were significantly higher than men (Table 3). 

**Table 3.** Comparing Mental Health Based on Independent *t*-test Groups Variable

	SD Average	Standard Error	*t*	Degree of Freedom	Level of Significance	Level of confidence 95%	
						Min	Max
**Anxiety (DASS)**							
**Gender**		1.20	- 0.58	47	0.56	- 3.11	1.711
Men	2.99 (3.41)						
Women	3.69 (4.78)						
**Education**		1.12	0.13	47	0.75	- 2.11	2.42
Lower than diploma	3.29 (3.57)						
Higher than diploma	3.14 (4.08)						
**Age**		1.24	2.06	36.45	0.04	0.04	4.41
Less than 40 years	4.19 (4.65)						
Over 40 years	1.95 (2.18)						
**Depression (DASS)**							
**Gender**		1.33	- 0.07	47	0.94	- 2.79	2.59
Men	6.30 (4.42)						
Women	6.39 (4.07)						
**Education**		1.23	1.38	47	0.55	- 0.77	4.17
Lower than diploma	7.33 (4.47)						
Higher than diploma	5.63 (3.23)						
**Age**		1.22	1.40	38.55	0.16	- 0.75	4.19
Less than 40 years	7.08 (4.59)						
Over 40 years	5.36 (3.23)						
**Stress (DASS)**							
**Gender**		1.66	- 0.27	47	0.78	- 3.80	2.90
Men	5.20 (5.23)						
Women	5.66 (5.70)						
**Education**		1.54	0.98	47	0.47	- 1.58	4.64
Lower than diploma	6.25 (5.32)						
Higher than diploma	4.72 (5.33)						
**Age**		1.45	2.49	36.89	0.01	0.67	6.56
Less than 40 years	7.12 (6.12)						
Over 40 years	3.50 (3.01)						
**Physical-insomnia disorders (GHQ)**							
**Gender**		1.39	- 2.03	16.59	0.05	- 5.77	0.10
Men	10.16 (2.33)						
Women	13.00 (5.15)						
**Education**		1.01	- 1.22	47	0.79	- 3.40	0.83
Lower than diploma	10.27 (3.13)						
Higher than diploma	11.55 (3.91)						
**Age**		1.15	0.92	39	0.32	-1.27	3.40
Less than 40 years	11.23 (4.02)						
Over 40 years	10.17 (2.82)						
**Anxiety disorders (GHQ)**							
**Gender**		1.62	- 1.17	17.78	0.25	- 5.32	1.49
Men	11.28 (3.22)						
Women	13.20 (5.90)						
**Education**		1.23	- 1.14	47	.053	- 3.88	1.07
Lower than diploma	11.03 (3.65)						
Higher than diploma	12.44 (4.59)						
**Age**		1.32	1.53	39	0.09	- 0.65	4.40
Less than 40 years	12.51 (4.78)						
Over 40 years	10.49 (2.74)						
**Socio-occupational disorders (DASS)**							
**Gender**		1.28	- 2.43	47	0.01	- 5.72	- 0.54
Men	12.20 (2.95)						
Women	15.34 (6.10)						
**Education**		1.25	- 1.49	47	0.57	- 4.39	0.64
Lower than diploma	12.05 (3.12)						
Higher than diploma	13.93 (4.95)						
**Age**		0.89	- 1.57	39	0.60	- 0.40	3.21
Less than 40 years	13.09 (2.67)						
Over 40 years	11.68 (2.98)						
**Severe depression (GHQ)**							
**Gender**		1.13	- 1.37	47	0.17	- 3.86	0.72
Men	8.63 (2.88)						
Women	10.20 (5.07)						
**Education**		1.09	0.06	47	0.75	- 2.11	2.26
Lower than diploma	7.33 (4.47)						
Higher than diploma	5.63 (4.06)						
**Age**		1.26	0.93	39	0.44	- 1.37	3.83
Less than 40 years	9.61 (3.75)						
Over 40 years	8.43 (4.22)						

## 5. Discussion

Investigation on mental health status of the staff of Tehran harm reduction centers showed that all of them suffered from mental health disorders, and almost 16% experienced serious levels of depression, anxiety and stress. Also, the levels of physical and socio-occupational disorders in women were significantly higher than fellow men. Besides, the results of the present study showed that the levels of anxiety and stress among staff younger than 40 years old were significantly higher than staff over 40 years old.

According to the 23 cut-off point (17) and Fathiashtiani et al. 100% of staff of harm reduction centers suffered from medium to high levels of disorders, and socio-occupational functioning disorder has the highest prevalence (65.1%) (16). According to Sahebi (20), the prevalence of mental disorders among nurses is high. But, comparing the results of the present study and those of Sadeghi et al. which was performed on hospital nurses -investigated physical disorders, anxiety, socio-occupational functioning and depression- the average score of the current study was three times higher than that of Sadeghi’s (21). Also, the staff of harm reduction centers had worse mental disorder than that of the staff of Behesht Zahra (S) organization (almost four times higher, according to the GHQ-28 questionnaire). In addition, participants of this study showed more mental disorders than student universities (22, 23). These findings indicated the need for considerable care and attention from responsible authorities (24). 

In the current study, mental health status of women staff was worse than that of fellow men. Asad Zandi et al. reported that psychological problems of women staff were more severe than those of men staff; a finding that is consistent with our present study (25). Regarding the causes of these problems, social effects of women’s attending the harm reduction centers should be noted. In this regard, Salehi et al. also showed that, among the people who referred to the facilities of the two different centers, “Medical Science University Headquarters” and “Education Department”, there is a significant difference between the mental health status of these two departments’ women staff (26). 

In this study, women showed more physical-insomnia and socio-occupational disorders. Mohammadian et al. (27) in their similar study showed that older and educated subjects had suffered more of these two mental health disorders. According to the current study, aging is one of the important factors affecting human mental health. Also, results of the studies by Pollack et al. (28) and Seyedan et al. (29) showed the higher level of anxiety and stress in the staff younger than 40 which is compatible with the results of the present study. According to the Shabani et al. (30) promotion is associated with job satisfaction.

This study showed that the staff of harm reduction centers suffered from considerable psychological problems. Accordingly, it seems reasonable that taking actions is necessary to improve the level of mental health in their staff. Thereby, the status of mental health in the staff of harm reduction centers (21), was significantly worse than that of nurses and staff of the Behesht Zahra (S) organization (24). The reason can lie in the relation between non-addicts and addicts that may leave destructive effects on non-addicts (8). This is more obvious in significantly lower mental health status of women staff compare to the fellow men.

Mental health differences between men and women staff of harm reduction centers are compatible with the findings of the previous researches who studied this subject from theoretical aspect, or in other words, socio-occupational disorders. Thus, female nurses suffer from mental health disorders more than men (25), and the similar results were found in the school students by Goodarzi and Roodbali (31). Also from practical aspect, and according to the cultural and religious conditions of the population under study, the presence of women in harm reduction centers may cause more problems for women staff regarding socio-occupational situations. 

Hence, it is necessary to consider more protective strategies for women staff in harm reduction centers, and preferably choose men for such jobs. Finally, Garousifarshi and Mani (32) showed that day shift staff suffered more from physical disorders and social problems than night shift workers. Finally, it may cause some differences among staff of harm reduction centers, and can be a subject for further researches.
